# Simultaneous Total Ankle Replacement and Contralateral Ankle Arthrodesis for Bilateral Ankle Osteoarthritis: A Retrospective Study Focused on Clinical Outcomes and Cost‐effectiveness

**DOI:** 10.1111/os.13390

**Published:** 2022-07-13

**Authors:** Jun Chen, Shizhou Wu, Yaxing Li, Yu Chen, Xi Liu, Boquan Qin, Hui Zhang

**Affiliations:** ^1^ Department of Orthopaedic Surgery, West China Hospital Sichuan University Chengdu People's Republic of China

**Keywords:** Ankle arthrodesis, Ankle osteoarthritis, Range of motion, Total ankle replacement

## Abstract

**Objective:**

Total ankle replacement (TAR) and ankle arthrodesis (AA) are two common surgical treatment options for end‐stage ankle osteoarthritis. However, few reports compare the outcomes of simultaneous TAR and contralateral AA for bilateral ankle osteoarthritis. The aim of this study was to assess changes in pain, joint range of movement (ROM), functional outcomes, patient satisfaction, and cost‐effectiveness following simultaneous TAR and contralateral AA.

**Methods:**

A retrospective study was conducted on 12 patients with bilateral end‐stage ankle osteoarthritis who underwent simultaneous TAR and contralateral AA in our institution between May 2016 and August 2018, and who had a minimum of two‐year follow‐up data. Clinical and radiological follow‐up data for all patients were collected after 4 months, 1 year and 2 years. The results were assessed clinically on a visual analogue scale (VAS) and included ROM, American Orthopedic Foot and Ankle Society (AOFAS) ankle hindfoot score, and satisfaction questionnaire. The total hospital costs of patients were also recorded. Independent sample *t* tests were conducted to compare continuous variables between groups. Paired sample *t*‐tests were conducted to compare changes from the preoperative to postoperative evaluations within each group.

**Results:**

Both surgical groups presented with pain reduction (*P* < 0.001) at the one‐year postoperative session, which was generally consistent until the two‐year follow‐up. There was a significant increase (*P* < 0.001) in the mean AOFAS score postoperatively in both ankles. The functional outcomes at the one‐ and two‐year follow‐up were significantly better in patients in the TAR group than in those in the AA group (*P* < 0.001). Joint ROM differences were observed between the two groups after surgery (decreased ankle ROM in arthrodesis, *P* < 0.001; increased ankle ROM in arthroplasty, *P* < 0.001). The mean satisfaction score was 2 (range, 1–4) for the TAR group and 3 (range, 1–5) for the AA group. A significant difference in the satisfaction score was observed between the two groups (*P* = 0.036). Simultaneous TAR and contralateral AA was 34.1% less expensive than simultaneous bilateral TAR. No intraoperative complications were noted in either group. Wound healing occurred without problems within 2 weeks after surgery. No symptomatic deep venous thrombosis was found during follow‐up.

**Conclusion:**

TAR had better patient‐perceived post‐operative function and preserves more anatomic sagittal plane motion compared to ankles undergoing AA. In addition, simultaneous TAR and contralateral AA are more cost‐effective than simultaneous bilateral TAR, with lower costs for the average patient.

## Graphical abstract

We found that TAR has better patient perceived post‐operative function and preserves more anatomic sagittal plane motion compared with patients undergoing AA. In addition, simultaneous TAR and contralateral AA is more cost‐effective than simultaneous bilateral TAR, with lower costs for the average patient.

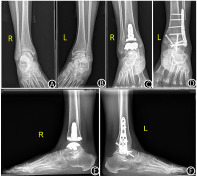

## Introduction

The incidence of osteoarthritis is lower for the ankle (approximately 6%) than for the knee or hip; however, end‐stage ankle osteoarthritis impacts the quality of life to a similar extent as osteoarthritis of larger joints.^1^, ^2^ Therefore, the treatment option is crucial to improve the quality of life. Total ankle replacement (TAR) and ankle arthrodesis (AA) are two common surgical treatment options for end‐stage ankle osteoarthritis.^1^, ^3^ The relative value of these alternative procedures is one of the most controversial issues in foot and ankle surgery.^4^, ^5^ To date, treatment selection depends on the experience and preference of the primary surgeon, as well as the preferences of the patient and family, with no evidence to assist in the decision. Severe osteoarthritis may occur in both ankles concurrently, causing patients to show symptoms sufficient to warrant bilateral TAR or AA.^6^, ^7^ Although previous studies have separately examined the effects of TAR and AA on pain, joint range of movement (ROM), and functional outcomes, limited research has directly compared the effects of TAR and AA.^4^, ^6^, ^8^ Moreover, few articles have assessed the outcomes following simultaneous TAR and contralateral AA.

Currently, the most frequent outcome measures used to evaluate the success of an operative intervention are outcome scales dependent on function and pain, such as the American Orthopedic Foot and Ankle Society (AOFAS) ankle hindfoot score,^9^ the Ankle Osteoarthritis Scale,^10^ and the Foot Function Index.^11^ In addition, most studies of TAR focus on revision rates, reoperation, complications and survivorship, while those of AA focus on re‐arthrodesis rates and complications. Patient satisfaction may be a valuable feedback for defining the success of operative intervention and useful to measure outcomes based on whether patients' exceptions to the operation have been met.^12^ The term “satisfaction” usually refers to the patient's perception of an orthopedic procedure and can be assigned to various attributes. For this study, the term was applied to either “satisfaction with operation” or to “satisfaction with symptoms.” In addition to indicating both relieved pain and improved joint function, satisfaction scores that quantify the extent to which a patient's exceptions have been met may reflect a patient's goals regarding the operation.

Although most of the patients were willing to accept simultaneous bilateral TAR, only 60% of the expenditure could be afforded because of the low earnings of the study cohort. To date, there have been no cost analyses of simultaneous TAR and contralateral AA compared to simultaneous bilateral TAR. Without the data on the outcomes and costs of simultaneous TAR and contralateral AA, it is crucial to provide quality, comparative effectiveness data to guide decision‐makers. Our priority is to provide patients with compassionate care while working to develop new treatments that will reduce the cost of treatment and offer more effective treatments.

To the best of our knowledge, few reports have compared the outcomes of simultaneous TAR and contralateral AA for bilateral ankle osteoarthritis. In addition, the knowledge concerning the satisfaction of patients with simultaneous TAR and contralateral AA is sparse. There have been no cost‐effectiveness analyses of simultaneous TAR and contralateral AA compared to simultaneous bilateral TAR. Therefore, we conducted a retrospective study to: (i) assess changes in pain, joint range of movement (ROM), functional outcomes and patient satisfaction after having had an ankle replaced or fused; (ii) present the main point of the operative techniques and the clinical outcomes; and (iii) evaluate the cost‐effectiveness of simultaneous TAR and contralateral AA compared to that of simultaneous bilateral TAR.

## Methods

### Inclusion Criteria

The inclusion criteria were as follows: (i) patients diagnosed with bilateral end‐stage ankle osteoarthritis at our hospital between May 2016 and August 2018; (ii) patients with a follow‐up period of ≥24 months; (iii) patients with well‐defined bilateral end‐stage ankle osteoarthritis who underwent simultaneous TAR and contralateral AA; and (iv) patients with well‐defined end‐stage ankle osteoarthritis who underwent AA.

### Exclusion Criteria

The exclusion criteria were as follows: (i) patients with poorly controlled diabetes or any confounding pathology; (ii) previous history of infectious arthropathy or AA; and (iii) patients without complete preoperative and postoperative follow‐up data.

### Patient Information

This study included 12 patients (24 ankles) who were diagnosed with bilateral end‐stage ankle osteoarthritis in our center from May 2016 to August 2018. Within our cohort, there were five males and seven females aged between 50 and 71 years (mean age 60 years). The reason for surgery was post‐traumatic arthritis in two (16.7%), primary osteoarthritis in seven (58.3%), and rheumatoid arthritis in three (25.0%). The research was approved by the Institutional Review Board of our institution (No. 2022‐588).

### Surgical Technique

#### Anesthesia and Position

Six patients had TAR on the left side and six had AA on the right side and *vice versa*. All surgeries were performed by a senior foot and ankle surgeon, with patients under general anesthesia in the supine position. The balloon of the proximal thigh tourniquet was inflated and the drapes were disinfected. The lower limbs and hip on the affected side were raised so that the toes pointed upward.

#### Approach and Operation

**Fig. 1:**
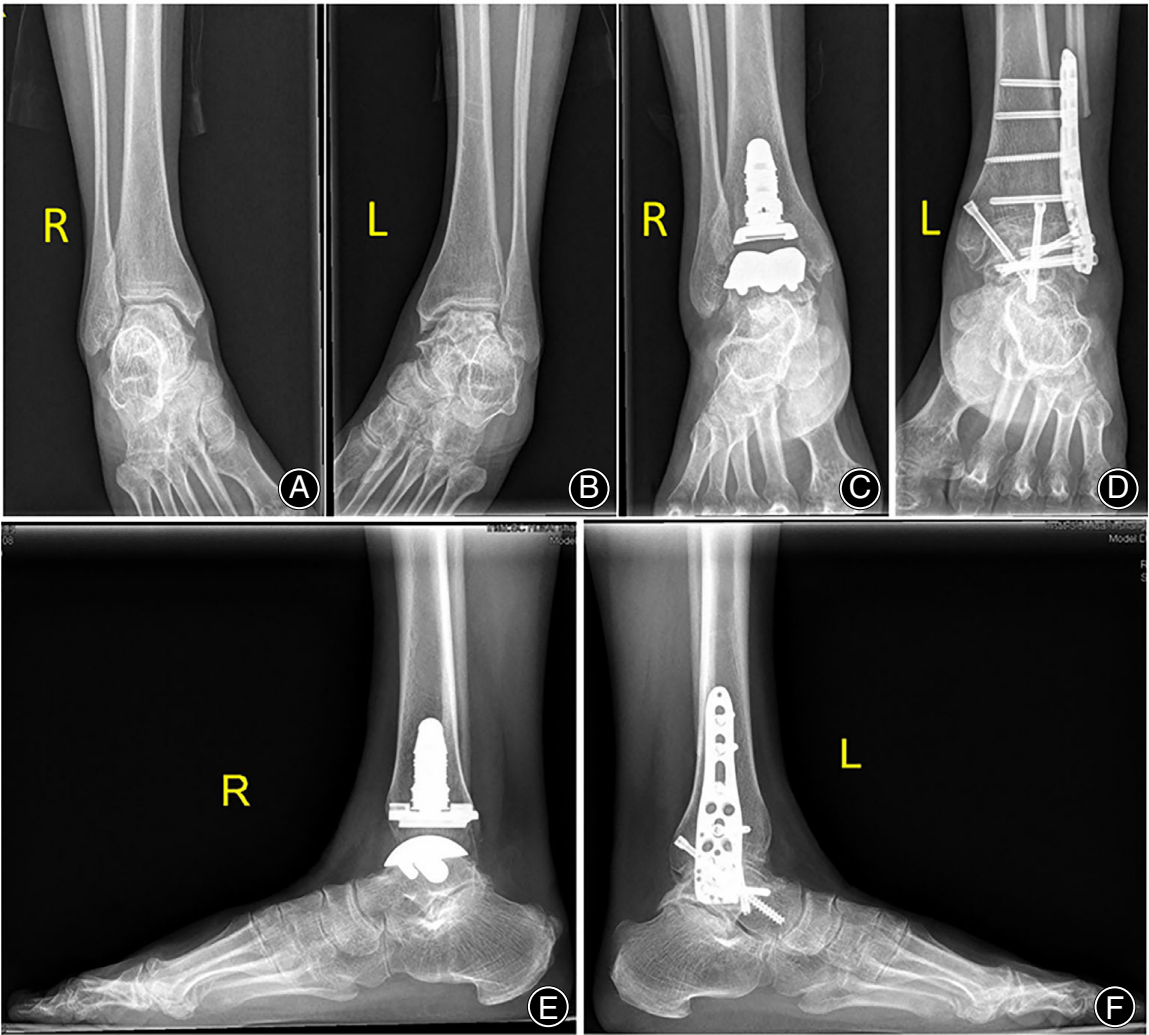
Reoperative and postoperative weightbearing radiographs of the arthroplasty and arthrodesis procedures. The patient was a 50‐year‐old female diagnosed with bilateral end‐stage ankle osteoarthritis (A, B). Anterior radiographs of INBONE II and arthrodesis (C, D). Lateral radiographs of INBONE II and arthrodesis (E, F)

**Fig. 2:**
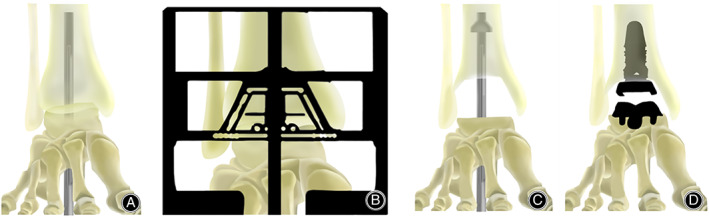
Total ankle replacement surgical diagrams of the key procedure. Creating a guide hole through the talus and calcaneus bones of the foot and into the base of the tibia (A). Extramedullary alignment and sizing for the tibial and talar cuts according to standard manufacturer's guidelines and techniques (B). Reshaping the end of the tibia and the top of the talus, and drilling a larger channel (C). Inserting the tibial and talar components (D)

The INBONE II TAR implant (Wright Medical Technology, Memphis, TN, USA) was used as a prosthesis (Fig. 1). A standard central anterior ankle incision was made using a scalpel. The interval between the extensor hallucis extensor tendon and the tibialis anterior tendon was used, with sharp dissection down through the ankle joint capsule, before executing an arthrotomy and exposing the ankle joint. At this moment, the lower limb was placed into the external fixation jig according to the manufacturer's guidelines and technique for the INBONE II TAR implant. All prostheses were placed using standard operative techniques.^13^ Implantation of the tibial, talar, and polyethylene components were performed successively. Final anteroposterior and lateral radiographs were taken intraoperatively. Surgical diagrams of TAR are shown in Fig. 2.

**Fig. 3:**
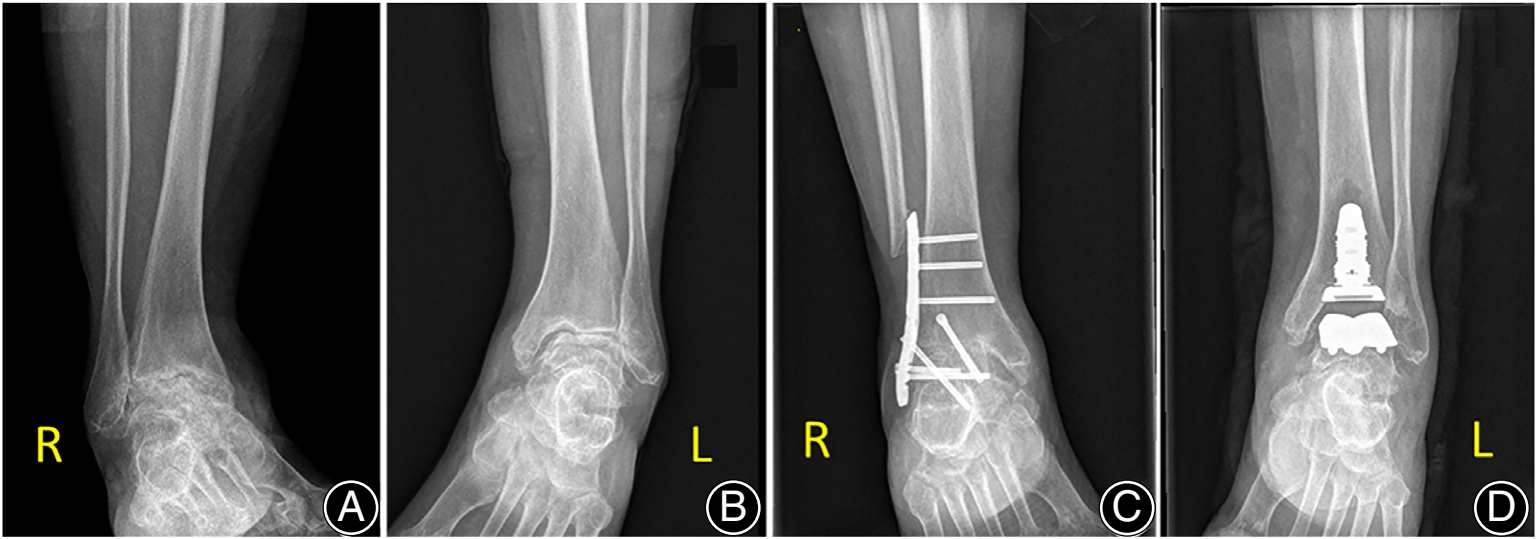
Preoperative and postoperative weightbearing radiographs of the arthroplasty and arthrodesis procedures. The patient was a 65‐year‐old female diagnosed with bilateral end‐stage ankle osteoarthritis (A, B). Anterior radiographs of INBONE II and arthrodesis (C, D)

**Fig. 4:**
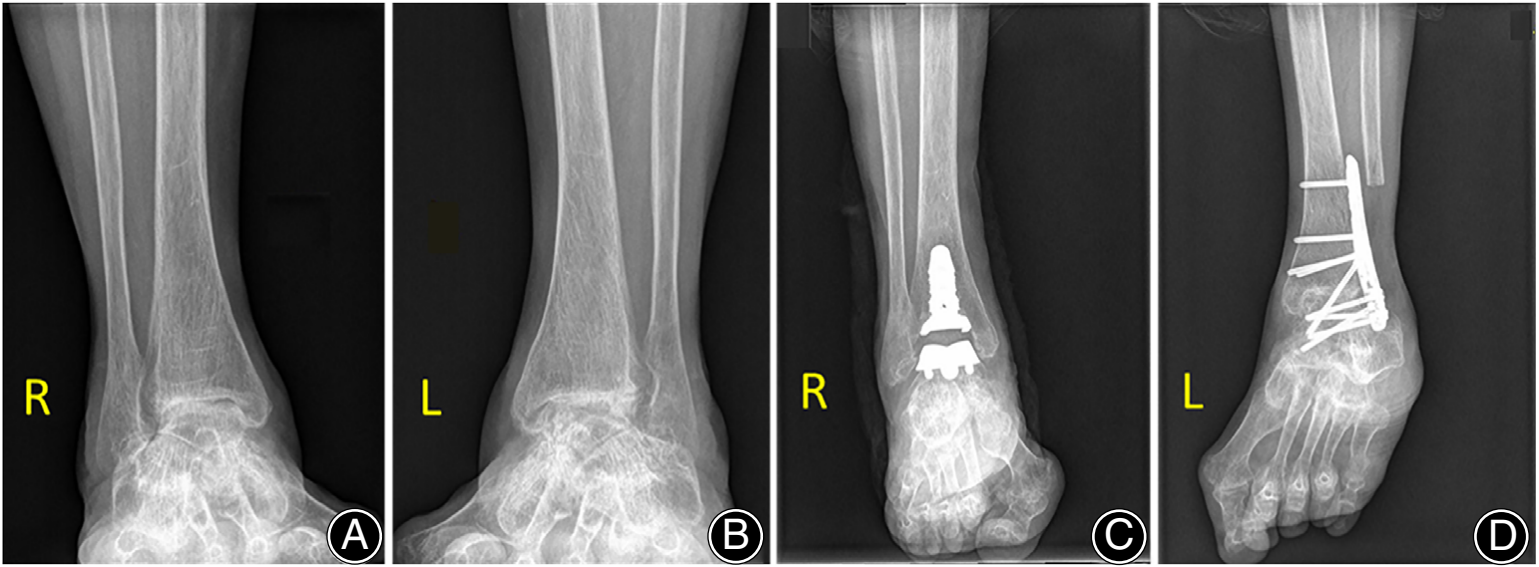
Preoperative and postoperative weightbearing radiographs of the arthroplasty and arthrodesis procedures. The patient was a 61‐year‐old female diagnosed with bilateral rheumatoid arthritis (A, B). Anterior radiographs of INBONE II and arthrodesis (C, D)

The AA was performed using reverse proximal humerus internal locking system (PHILOS) plating with cannulated screwing (Changzhou Dingjian Medical Appliance Co., Ltd., Changzhou, China) *via* the transfibular approach (Fig. 1). A longitudinal skin incision was made, beginning 8 cm proximal to the tip of the lateral malleolus and continuing along the posterior border of the fibula in a distal direction. The subcutaneous and fascial layers were cut sequentially to expose the distal fibula. At a distance of approximately 6 cm from the distal end of the fibula, a pendulum saw was applied to cut off the oblique fibula and remove the distal end of the fibula cancellous bone surface. At this time, the removal cartilage surface together with the subchondral plate curettage, the bone was cut, and soft tissue was loosened to correct any deformity and alignment. PHILOS plate reserve and applied to the tibia bone, proximally fixed to the talus with a cannulated screw. Examples of preoperative and postoperative radiographs are provided for both procedures (Figs 3 and 4).

#### Postoperative Management

Low molecular weight heparin was used to prevent deep venous thrombosis after surgery. Postoperatively, patients who underwent TAR were placed in a short leg splint and were required to be non‐weight‐bearing for 2 weeks. For the next 3 weeks, ankle ROM exercise was recommended while maintaining the short leg splint. A foot and ankle rehabilitation program, which included proprioceptive exercise, calf strengthening and stretching of the triceps surae, and full weight‐bearing were started 8 weeks postoperatively. Postoperatively, patients who underwent AA were placed in a short‐leg cast and were required to be non‐weight‐bearing for 2 weeks. A foot and ankle rehabilitation program, which included proprioceptive exercise, calf strengthening and stretching of the triceps surae, and full weight‐bearing were started 8 weeks postoperatively.

#### Outcome Evaluation

Clinical and radiological follow‐up data for all patients were conducted after 4 months, 1 year, and 2 years. The outcome measures were the preoperative and postoperative American Orthopedic Foot and Ankle Society (AOFAS) hindfoot score, ROM (dorsiflexion, plantarflexion), visual analogue scale (VAS) and satisfaction scores. Meanwhile, complications that occurred during surgery and post‐operation, operation time and the total hospital costs of patients were also recorded.

#### American Orthopedic Foot and Ankle Society Hindfoot Score

The American Orthopedic Foot and Ankle Society hindfoot score **(**AOFAS) hindfoot score was used to measure the treatment outcome in patients who had sustained a complex ankle or hindfoot injury. The maximum value of the AOFAS ankle hindfoot score was 100 points. The AOFAS hindfoot score system can be classified into three subscales, including pain (40 points), function (50 points), and alignment (10 points). The maximum scores of pain, function, and alignment indicate painless, full function, and good alignment, respectively.

#### ROM Measurement

The angle of maximum passive ankle plantarflexion and dorsiflexion were measured by a goniometer before and after surgery. An independent observer, who was a physical therapist or a trained medical provider, performed all measurements. The ankle ROM was measured using a goniometer along the long axis of the tibia and a line horizontal to the weight‐bearing surface to determine ankle plantarflexion and dorsiflexion.^8^

#### Visual Analogue Scale

Ankle pain is an important symptom and a frequent patient complaint. A self‐reported score on the 10‐point VAS is used in the social and behavioral sciences to measure a patient's degree of pain. All patients reported their pre‐ and postoperative pain on a scale of 0 to 10, where 0 indicates no pain, and 10 indicates extreme pain.

#### Satisfaction Scores

During the follow‐up, all patients reported their satisfaction with the result of each ankle according to a five‐grade Likert scale as follows: satisfied, satisfied, neither satisfied nor dissatisfied, dissatisfied, and very dissatisfied,^14^ where very dissatisfied corresponds to 5 points and satisfied to 1 point.

### Statistical Analysis

SPSS version 19.0 (IBM Corp., Armonk, NY, USA) was used to perform statistical analysis. The Shapiro–Wilk test was used to investigate whether the measurement data were normally distributed, including the AOFAS ankle hindfoot score, preoperative and postoperative ROM, VAS score and satisfaction score. Independent sample *t*‐tests were conducted to compare continuous variables between the TAR and contralateral AA. Paired sample *t*‐tests were conducted to compare changes from the preoperative to the postoperative evaluations within each group. Statistical significance was defined as a *P* value <0.05.

## Results

### General Surgical Outcomes

The average operative time in the TAR group was 110 min (range, 90 to 130 min) and the average operative time in the AA group was 50 min (range, 45 to 65 min). The total cost was 201,522 RMB yuan for simultaneous bilateral TAR compared to 132,822 RMB yuan for simultaneous TAR and contralateral AA. Simultaneous TAR and contralateral AA were 34.1% less expensive than simultaneous bilateral TAR. Clinical and radiological parameters were recorded for each patient before and after surgery at 4 months, 1 year, and 2 years (Tables 1 and 2). Before surgery, the two groups were not significantly different with regard to the average pain scores (*P* = 0.878) (Table 1). Similarly, the mean ROM (*P* = 0.849) and AOFAS ankle hindfoot score (*P* = 0.854) were comparable in both groups (Table 2).

**TABLE 1:** Level of pain (VAS^a^) pre‐and post‐operation according to group

	TAR^b^	Fusion	*t*‐value	*P*‐value^c^
Pre‐operative				
Starting pain	6.5 (4 to 8)	6.4 (4 to 9)	0.144	0.886
Stress pain	8.6 (7 to 10)	8.7 (6 to 10)	0.175	0.863
Pain at rest	3.0 (1 to 5)	2.9 (1 to 5)	0.192	0.850
Pain at night	3.4 (2 to 6)	3.5 (1 to 6)	0.332	0.743
Overall pain	7.5 (5 to 9)	7.4 (5 to 9)	0.155	0.878
4‐month follow‐up				
Starting pain	2.2 (0 to 4)	1.9 (0 to 3)	0.604	0.552
Stress pain	3.7 (2 to 5)	3.6 (2 to 5)	0.243	0.811
Pain at rest	1.3 (0 to 3)	1.2 (0 to 3)	0.215	0.832
Pain at night	1.5 (0 to 3)	1.4 (0 to 3)	0.240	0.813
Overall pain	2.7 (2 to 4)	2.4 (1 to 4)	1.176	0.252
1‐year follow‐up				
Starting pain	1.2 (0 to 3)	1.3 (0 to 3)	0.226	0.823
Stress pain	2.6 (1 to 4)	2.2 (1 to 4)	1.176	0.252
Pain at rest	1.1 (0 to 3)	1.0 (0 to 3)	0.209	0.836
Pain at night	1.2 (0 to 3)	1.1 (0 to 3)	0.211	0.835
Overall pain	1.5 (0 to 4)	1.4 (0 to 3)	0.233	0.818
2‐year follow‐up				
Starting pain	1.3 (0 to 3)	1.1 (0 to 3)	0.437	0.666
Stress pain	1.9 (0 to 4)	1.4 (0 to 3)	1.176	0.252
Pain at rest	1.1 (0 to 3)	1.0 (0 to 3)	0.209	0.836
Pain at night	1.1 (0 to 3)	1.0 (0 to 3)	0.209	0.836
Overall pain	1.3 (0 to 3)	1.2 (0 to 3)	0.240	0.813

Data are presented as a mean (range).

^a^ VAS, visual analogue scale from 0 (no pain) to 10 (maximum pain).

^b^ TAR, total ankle.

^c^ Replacement using independent‐sample t‐tests.

**TABLE 2:** Range of movement (degrees) of ankle joint and American Orthopedic Foot and Ankle Society (AOFAS) hindfoot score before and after surgery according to group

	TAR^a^	Fusion	*t*‐value	*P*‐value^b^
Pre‐operative				
Total motion arc	27.3 (17 to 40)	26.7 (18 to 43)	0.193	0.849
Dorsiflexion	2.5 (−5 to 6)	2.7 (−4 to 8)	0.130	0.897
Plantarflexion	24.8 (15 to 35)	24.0 (15 to 35)	0.304	0.764
AOFAS score	35.0 (22 to 50)	35.8 (23 to 49)	0.186	0.854
4‐month follow‐up				
Total motion arc	40.5 (32 to 52)	13.5 (10 to 19)	0.058	< 0.001
Dorsiflexion	11.3 (8 to 15)	0.6 (−3 to 4)	0.587	< 0.001
Plantarflexion	29.2 (22 to 40)	12.9 (9 to 18)	0.213	< 0.001
AOFAS score	79.2 (70 to 85)	72.6 (66 to 79)	0.395	0.001
1‐year follow‐up				
Total motion arc	41.1 (33 to 52)	13.9 (10 to 19)	0.074	< 0.001
Dorsiflexion	11.6 (8 to 15)	0.8 (−2 to 4)	0.159	< 0.001
Plantarflexion	29.5 (22 to 40)	13.1 (9 to 18)	0.332	< 0.001
AOFAS score	85.1 (76 to 90)	80.2 (74 to 87)	0.253	0.009
2‐year follow‐up				
Total motion arc	41.2 (33 to 52)	13.8 (10 to 19)	0.069	< 0.001
Dorsiflexion	11.6 (8 to 15)	0.8 (−2 to 4)	0.159	< 0.001
Plantarflexion	29.6 (22 to 40)	13.0 (9 to 18)	0.369	< 0.001
AOFAS score	85.5 (77 to 90)	81.0 (74 to 88)	0.101	0.014

Data are presented as a mean (range).

^a^ TAR, total ankle replacement;

^b^ Using independent ‐sample t‐tests

### American Orthopedic Foot and Ankle Society Hindfoot Score

There was a significant increase (*P* < 0.001) in the mean AOFAS scores postoperatively in both ankles. In addition, the functional outcome at the 1‐ and 2‐year follow‐up were significantly better in patients in the TAR group than in those in the AA group (*P* < 0.001).

### Range of Movement

**Fig. 5:**
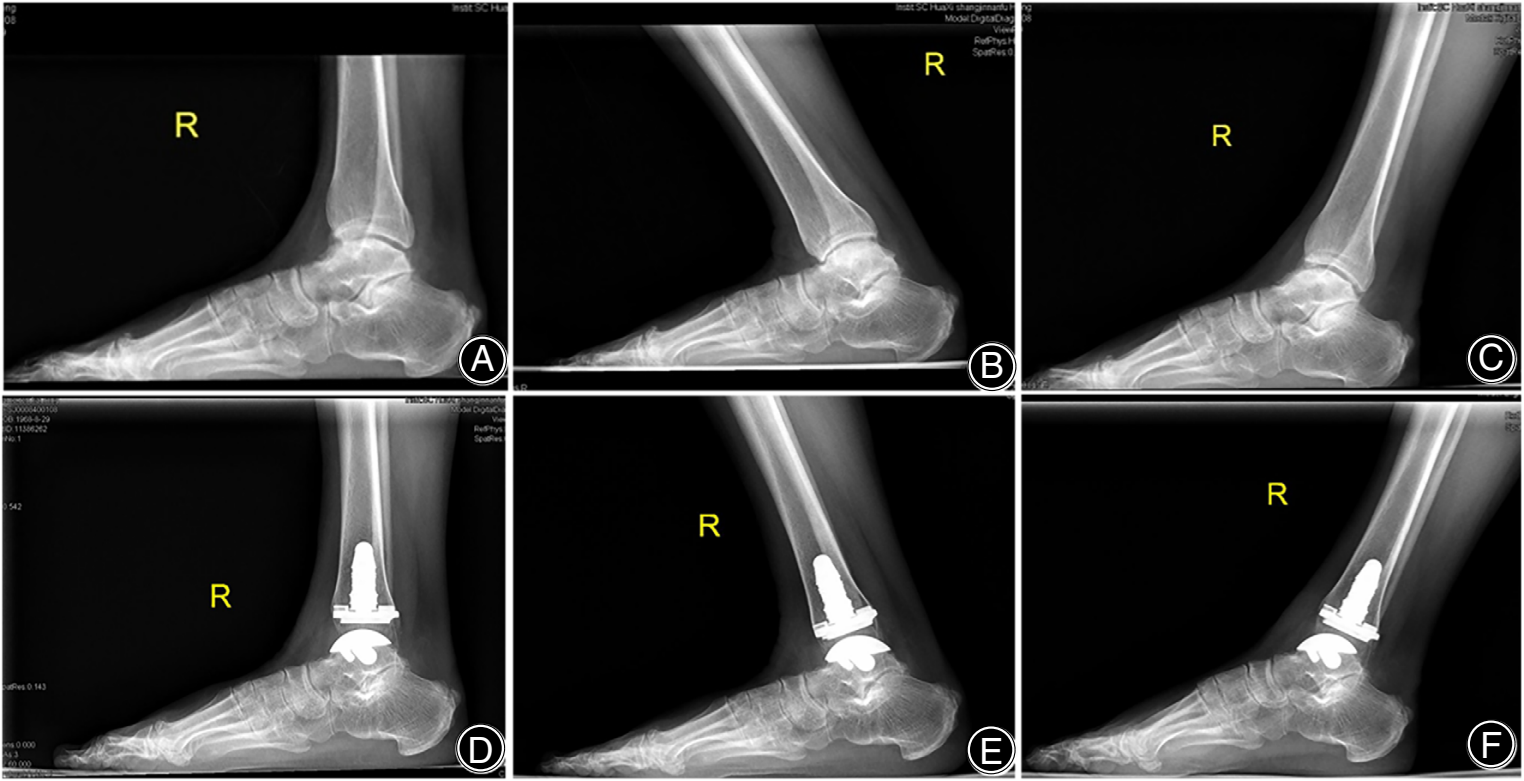
Two‐year follow‐up of a 50‐year‐old female with right total ankle replacement and satisfactory clinical outcome. Preoperative lateral weightbearing view (A). Preoperative lateral weightbearing dorsiflexion and plantarflexion views (B, C). Postoperative lateral weightbearing view (D). Postoperative lateral weightbearing dorsiflexion and plantarflexion views (E, F)

Patients who underwent TAR maintained a greater average total sagittal movement than patients who underwent AA (Fig. 5). Radiographic assessment of the four‐month follow‐up ankle ROM demonstrated a significant difference between the two groups with respect to dorsiflexion, plantarflexion, and total motion arc (Table 2). The TAR group showed an increased ankle ROM (from 27.3° to 40.5°) (*P* < 0.001), with improved dorsiflexion and plantarflexion (Table 2). The AA group exhibited decreased ankle ROM (from 26.7° to 13.5°), with equal decreases occurring in dorsiflexion and plantarflexion (Table 2). After the four‐month follow‐up, all patients treated with simultaneous TAR and contralateral AA had restored ability for daily activities.

### Visual Analogue Scale

Both surgical groups presented pain reduction (*P* < 0.001) at the four‐month postoperative session, which was generally consistent across the two‐year follow‐up. At the four‐month follow‐up, patients in the AA group reported a lower mean pain score (2.2 [0 to 4]) than those in the TAR group (1.9 [0 to 3]), although no significant difference in pain score was observed between the two groups (*P* = 0.324) (Table 1).

### Satisfaction Scores

The mean satisfaction score was 2 (range, 1–4) for the TAR group and 3 (range, 1–5) for the AA group. A significant difference in the satisfaction score was observed between the two groups (*P* = 0.036).

### Complication and Revision

No intraoperative complications were noted in either group. Wound healing occurred uneventfully within 2 weeks after surgery. No symptomatic deep venous thrombosis was found during follow‐up. There was no revision in either group during the two‐year follow‐up.

## Discussion

### Study Summary

TAR and AA are two major surgical treatment options for end‐stage ankle osteoarthritis, and their relative advantages remain controversial issues in foot and ankle surgery.^4^, ^5^ However, limited information is available to guide the surgeon and patient regarding the influence of bilateral end‐stage ankle arthritis and the treatment options, such as simultaneous TAR and contralateral AA. Other authors have sporadically reported simultaneous bilateral TAR or AA for ankle osteoarthritis.^7^, ^15^ However, few studies have assessed the outcomes following simultaneous TAR and contralateral AA. Therefore, we reviewed such patients from our institution over 2 years to assess changes in pain, joint ROM, functional outcomes, patient satisfaction, and cost‐effectiveness.

The key to successful execution can be divided into three periods: preoperative, intraoperative, and postoperative. During the preoperative phase of patient selection, particular attention should be directed at the expectations, age, body habitus, activity demands, and soft tissue envelope. The techniques and basic principles of TAR are generally standardized among most major surgical implants. However, some subtle technical alterations during the operation can contribute to ensure more predictable outcomes while minimizing complications. Previous studies have shown that TAR with the prosthesis is technically challenging and there exists a learning curve, whereby increased surgeon experience and careful patient selection can decrease failure rates and improve outcomes.^16^, ^17^ In addition, postoperative nursing, and correct, effective physical exercises are the key steps of the operative success and ensuring the patients convalescence.

In this study, patients presenting with the bilateral disease were diagnosed primarily with post‐traumatic arthritis (16.7%), primary osteoarthritis (58.3%), and rheumatoid arthritis (25.0%). Previous studies have asserted that bilateral end‐stage ankle osteoarthritis is most prevalent in primary osteoarthritis.^18^ Our study is consistent with these previous results. We found no complications associated with the length of operative time, such as anesthetic, blood loss, and medical complications, and all of the patients achieved wound healing within 2 weeks of surgery. No symptomatic deep venous thrombosis was found during follow‐up. Although thromboembolism is not recorded as a common complication following TAR,^19^ previous studies have included some form of prophylactic regime. Routine deep vein thrombosis prophylaxis is recommended for any lower limb replacement procedure, especially in the context of prolonged operation time and the application of bilateral tourniquets.

### Benefits and Costs of Simultaneous TAR and Contralateral AA

In a previous study, bilateral AA was recognized as a challenging treatment given its impairment of function.^7^ Patients who underwent bilateral AA were noted to have great difficulty with inclines, stairs, and walking on uneven terrain. Simultaneous bilateral arthroplasty of the knee and hip has been documented as a good alternative to staged procedures,^20^, ^21^, ^22^ with benefits including shorter total recovery time, single anesthetic, and significantly reduced overall hospital stay and costs.^20^, ^21^, ^22^, ^23^, ^24^ Compared to simultaneous bilateral TAR, simultaneous TAR and contralateral AA has the advantages of shorter operation time and low cost. However, this balance also relies on the local reimbursement policy by Medicare. Our findings demonstrate that simultaneous TAR and contralateral AA are more cost‐effective than simultaneous bilateral TAR, with lower costs for the average patient.

### Clinical Scores and Ankle ROM after Surgery

Ankle pain is the most common symptom of end‐stage ankle osteoarthritis. The primary benefit of TAR or AA is to relieve pain. In our series, both surgical groups presented with pain reduction (*P* < 0.001) at the four‐month postoperative session, which was generally consistent across the two‐year follow‐up. At the four‐month follow‐up, patients in the AA group reported a lower mean pain score than those in the TAR group, but no significant difference in pain score was observed between the two groups (*P* = 0.324). A recent meta‐analysis showed that there was still no significant difference in postoperative pain relief between TAR and AA after upgrading clinical outcomes obtained from modern techniques.^3^ Our findings are consistent with this result.

ROM at the ankle joint is mainly dorsiflexion and plantarflexion in the sagittal plane. In this study, joint ROM differences were observed between the two groups after surgery. The TAR group showed an increased ankle ROM, with improvement occurring in dorsiflexion and plantarflexion. However, the AA group exhibited a decreased ankle ROM, with equal decreases occurring in dorsiflexion and plantarflexion; these findings are consistent with those of a previous study.^3^ Moreover, the effect on gait is reported to be larger after AA compared to TAR,^25^ even if the gait ability does improve after both TAR and AA.^26^ The movement in the AA group arose from compensatory hypermobility at the adjacent midfoot joints. A previous study demonstrated that the level of activity did not significantly differ between TAR and AA.^27^ In our study, all patients treated with simultaneous TAR and contralateral AA had restored ability for daily activities, suggesting that this therapy could be chosen depending on the preoperative level of social functioning.

A recent meta‐analysis showed that the TAR group had a significant improvement in functional outcome compared to the AA group.^3^ In this study, there was a significant increase (*P* < 0.001) in the mean AOFAS score postoperatively in both ankles. In addition, the functional outcome at the 1‐ and two‐year follow‐up were significantly better in patients in the TAR group than in those in the AA group (*P* < 0.001).

### Satisfaction

Patient satisfaction may be valuable feedback for defining the success of an operative intervention.^12^ In the present study, the mean satisfaction score was 2 (range, 1–4) for the TAR group and 3 (range, 1–5) for the AA group. A significant difference in the satisfaction score was observed between the two groups (*P* = 0.036). Younger *et al*.^28^ reported that the improvement in satisfaction with clinical symptoms was greater for the TAR group, although this operation mode had a considerably higher revision rate. Our study suggests that good outcomes can be obtained with simultaneous TAR and contralateral AA and is a good alternative to bilateral end‐stage ankle osteoarthritis. However, in certain situations, bilateral TAR is contraindicated and clinicians are therefore limited in their selections. Currently, there is limited information that surgeons can provide to their patients on the outcomes of simultaneous TAR and contralateral AA in these settings. Our effective treatment regime has yielded satisfactory results in patients with bilateral ankle osteoarthritis suffering no major postoperative complications. These patients achieved good postoperative function and pain relief, as well as excellent satisfaction relating to the surgeon experience.

### Limitations

This study had several limitations, including the small number of patients with bilateral end‐stage ankle osteoarthritis and the short follow‐up. However, the condition with simultaneous TAR and contralateral AA was unusual. Previous studies have revealed that the incidence of patients with bilateral end‐stage ankle osteoarthritis is thought to be much lower in their respective cohorts.^29^, ^30^ A further limitation of the study was its retrospective design. Despite these limitations, we believe that our results have important contributions to describing the functional outcomes of patients who have undergone simultaneous TAR and contralateral AA. We will continue to follow up on the medium‐ and long‐term outcomes of simultaneous TAR and contralateral AA for bilateral ankle osteoarthritis.

### Conclusion

We found that TAR had better patient‐perceived post‐operative function and preserved more anatomic sagittal plane motion compared to that observed in patients undergoing AA. Simultaneous TAR and contralateral AA were more cost‐effective than simultaneous bilateral TAR, with lower costs for the average patient. This treatment regime yielded satisfactory results, with bilateral ankle osteoarthritis patients suffering no major postoperative complications. We considered simultaneous TAR and contralateral AA to be a reasonable treatment for patients with bilateral end‐stage ankle arthritis when no other treatment option was available.
